# Pars Plana Vitrectomy for Inadvertent Intravitreal Injection of Hyaluronic Acid Dermal Filler: A Case Report

**DOI:** 10.1159/cop/acaag005

**Published:** 2026-08-18

**Authors:** Chloe J. Cohan, Jordan M. Graff

**Affiliations:** a Creighton University School of Medicine, Phoenix, AZ, USA; b Barnet Dulaney Perkins Eye Center at American Vision Partners, Phoenix, AZ, USA

**Keywords:** Dermal filler, Hyaluronic acid, Juvéderm, Vitreous cavity, Vitrectomy, Laser retinopexy, Oculoplastics

## Abstract

**Introduction:**

Hyaluronic acid (HA) dermal fillers are commonly used in cosmetic practice and are generally safe; however, ocular complications may occur if material is introduced into periocular or intraocular structures. Although vascular-related vision loss has been widely reported, direct intravitreal dermal filler deposition is exceedingly rare, and subsequent management with vitrectomy has not been previously documented. This report presents the clinical course and management of inadvertent intravitreal injection of HA filler and highlights considerations for safe removal.

**Case Presentation:**

A 47-year-old woman experienced immediate, transient vision loss following temporal filler injection with HA-based cosmetic filler (i.e., Juvéderm Voluma), with partial spontaneous recovery within minutes. She presented days later with persistent blurred vision and a mobile opacity within the vitreous cavity. Multimodal imaging demonstrated a scintillating intravitreal deposit consistent with HA filler. After initial management with topical corticosteroids and close observation, worsening visual acuity and persistent blurred vision prompted prophylactic puncture site laser retinopexy and uncomplicated 25-gauge pars plana vitrectomy. Postoperatively, visual acuity improved to 20/20, intraocular pressure remained stable, and retinal architecture was preserved without evidence of inflammation or toxicity.

**Conclusion:**

This case highlights a rare mechanism of ocular injury related to cosmetic filler injection. The highly cross-linked nature of modern HA fillers likely limits spontaneous degradation within the vitreous, making surgical removal a reasonable consideration in symptomatic patients. Prompt recognition and appropriate ophthalmic referral are essential. While primary prevention of such complications is paramount, pars plana vitrectomy can be safely and effectively performed to restore vision and mitigate long-term retinal risk in select cases.

## Background and Introduction

Hyaluronic acid (HA) dermal filler injections are among the most commonly performed nonsurgical cosmetic procedures in the USA, used to restore facial volume, smooth wrinkles, and enhance contours [1, 2]. Their popularity reflects versatility, relatively favorable safety profiles, predictable aesthetic outcomes, and reversibility with hyaluronidase [3–5]. Although generally safe, adverse events can occur, particularly in the periorbital region, where inadvertent injection may lead to ocular complications.

HA is a naturally occurring glycosaminoglycan present in skin, synovial fluid, connective tissue, and vitreous [6, 7]. In widely used fillers such as Juvéderm Voluma, HA is extensively cross-linked to enhance stability, longevity, and resistance to degradation [8, 9].

Ocular sequelae from dermal filler injections most commonly result from vascular events, in which retrograde arterial flow can embolize the ophthalmic or retinal circulation, leading to sudden and often irreversible vision loss [10–12]. Reported manifestations include retinal artery occlusion, ischemic optic neuropathy, and stroke [11, 12]. These complications occur most frequently in areas with dense vascular anastomoses, including the glabella, nose, nasolabial folds, forehead, and temples [11, 13].

Vascular complications occur in approximately 0.01–0.05% of injections, with higher rates reported using needles than cannulas, and nearly half of affected patients experiencing permanent vision loss [14–16]. Prompt recognition, cessation of injection, and urgent ophthalmologic consultation are essential; if HA is implicated, hyaluronidase may be attempted, though visual recovery remains unpredictable [4, 5, 17, 18].

While vascular complications are well-documented, reports of direct intravitreal filler deposition are exceedingly rare, with no prior reports describing surgical removal of intravitreal HA as definitive management [19]. This case represents a novel complication with important implications for ophthalmic practice, highlighting the potential role of pars plana vitrectomy for visual recovery and retinal preservation.

## Case Presentation and Initial Management

Our patient is a healthy 47-year-old female who presented 3 days after receiving HA filler (Juvéderm Voluma) for temporal hollowing. A cannula was not used; the injector did not document specific needle gauge or aspiration technique. During injection into the left temple, the patient experienced left periorbital pain and near-immediate vision loss to light perception only, prompting the injector to stop and withdraw the needle after approximately 0.5–1 cc of filler was delivered. Hyaluronidase was then injected into the same region of skin, and the area massaged; vision partially returned within 8–10 min but remained blurry.

On presentation to the general ophthalmology clinic, the physician suspected ocular puncture and referred the patient to our retina team. Injection documentation was reviewed. Per initial history, the patient described her vision as like looking “through a mirage” or “through a chain-link fence.” Her best-corrected visual acuity (BCVA) was 20/30, and intraocular pressure (IOP) was 10 mm Hg. Slit lamp examination revealed only mild conjunctival inflammation with no anterior segment cells. Scleral depressed retinal examination was performed, as well as fluorescein angiography to assess retinal and posterior segment findings (Fig. 1, 2). There was no retinal filling defect, whitening, edema, leakage, or hemorrhage.

**Fig. 1.**
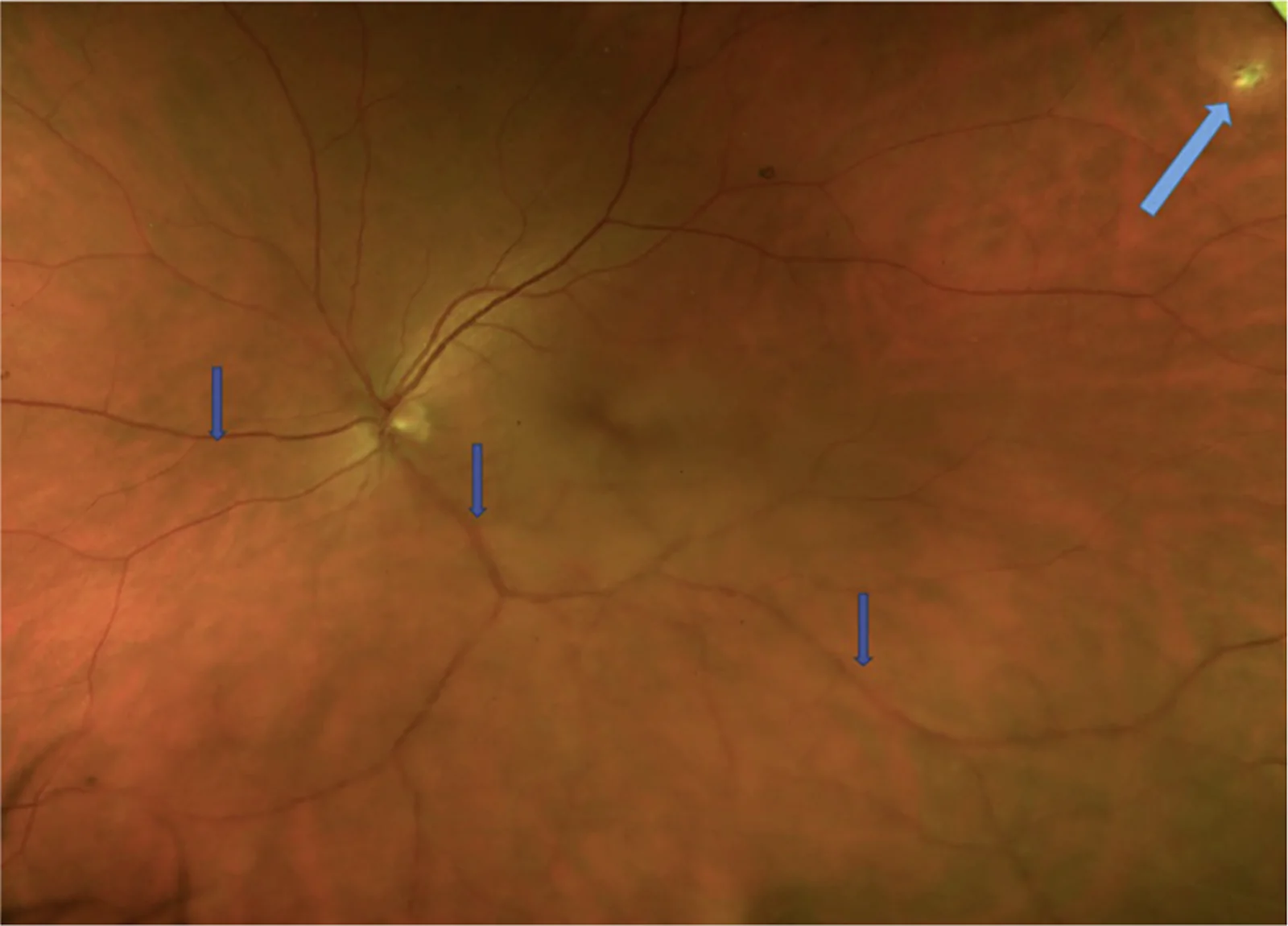
Initial fundus imaging demonstrating retinal entry site and intravitreal filler. Retinal imaging on first visit after accidental intravitreal dermal filler injection. Note singular retinal puncture site (light blue arrow) and the opaque mirage effect on the view of the retina through the foreign intravitreal material (dark blue arrows).

**Fig. 2.**
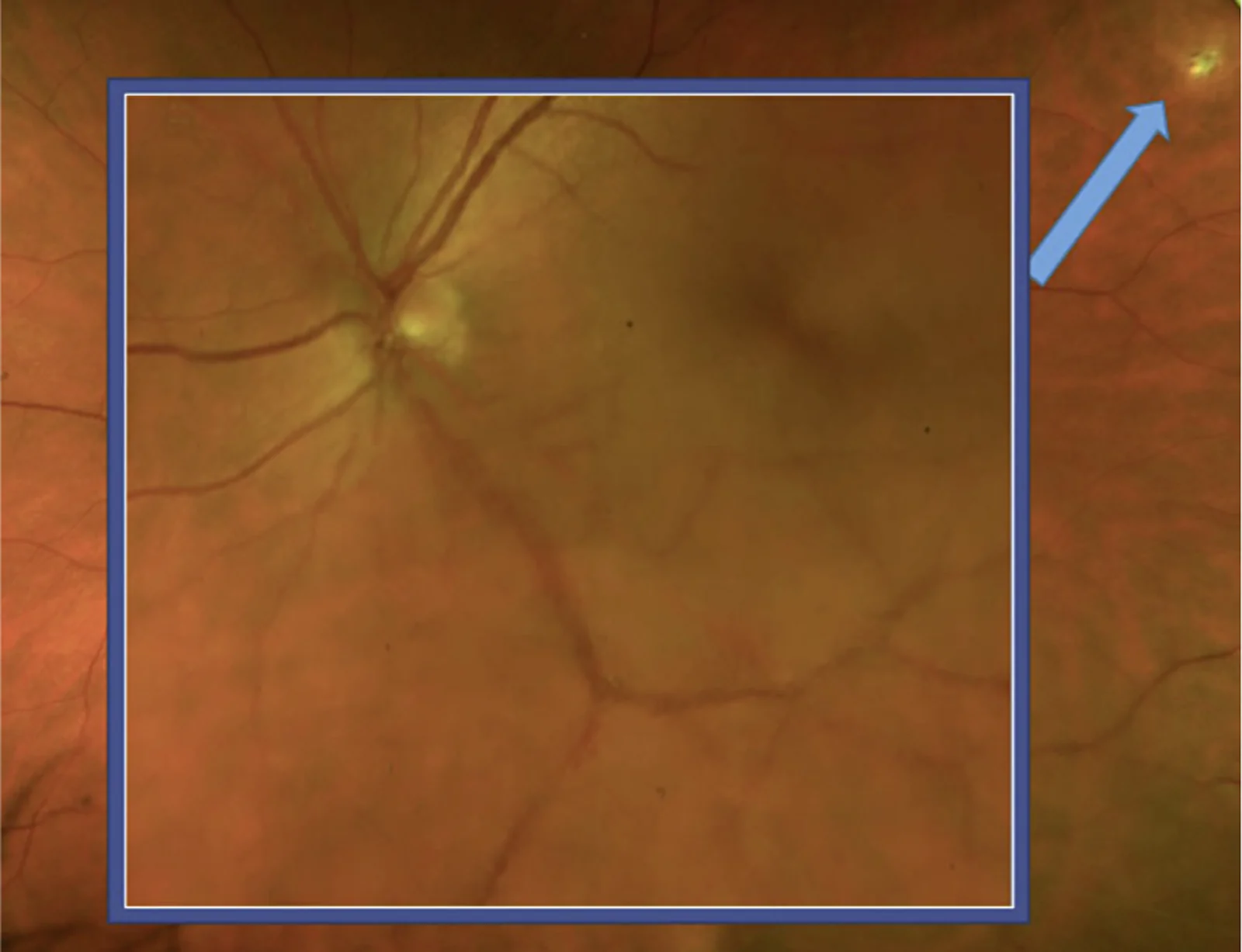
Magnified initial fundus view. Zoomed-in view of retinal imaging revealing blurring of underlying retinal vessels, suggesting presence of HA filler in vitreous. Light blue arrow re-demonstrates needle puncture site.

As an early precaution throughout the initial observation period and while definitive management options were being considered, in-office laser photocoagulation was applied around the needle puncture site to minimize risk of adjacent retinal tear or detachment. Additionally, prednisolone acetate ophthalmic drops and artificial tears were initiated to address mild conjunctival inflammation and irritation.

Management options included observation versus pars plana vitrectomy for filler removal. We emphasized that observation would not preclude future vitrectomy if the patient’s condition failed to improve or worsened. Potential surgical risks associated with any vitrectomy (i.e., retinal detachment, endophthalmitis, and early-onset cataract) were discussed. The potential for unknown risks in removing cross-linked HA from the vitreous cavity was discussed as well, as the material’s intraoperative behavior and need for larger-gauge access for removal were uncertain. The patient elected initial observation to assess for spontaneous symptom improvement and to avoid surgical risk in the absence of retinal traction or inflammation, with the understanding that vitrectomy could be pursued promptly if visual function worsened.

One week later, BCVA in the left eye had worsened to 20/400, while IOP remained 10 mm Hg. The patient reported persistent visual disturbances, including a mobile floating “glob” and intermittent “mirage”-like distortions with variable acuity. Repeat examination and retinal imaging at this time showed persistence of the HA in the vitreous, now opacifying the visual axis (Fig. 3). Retinal architecture remained intact, with no evidence of a new retinal break, detachment, or inflammatory response. The decision was made to perform a vitrectomy to remove the HA filler, with the goals of improving vision and preventing potential complications of retained material, including persistent visual disturbance, inflammation, retinal toxicity, and retinal traction.

**Fig. 3.**
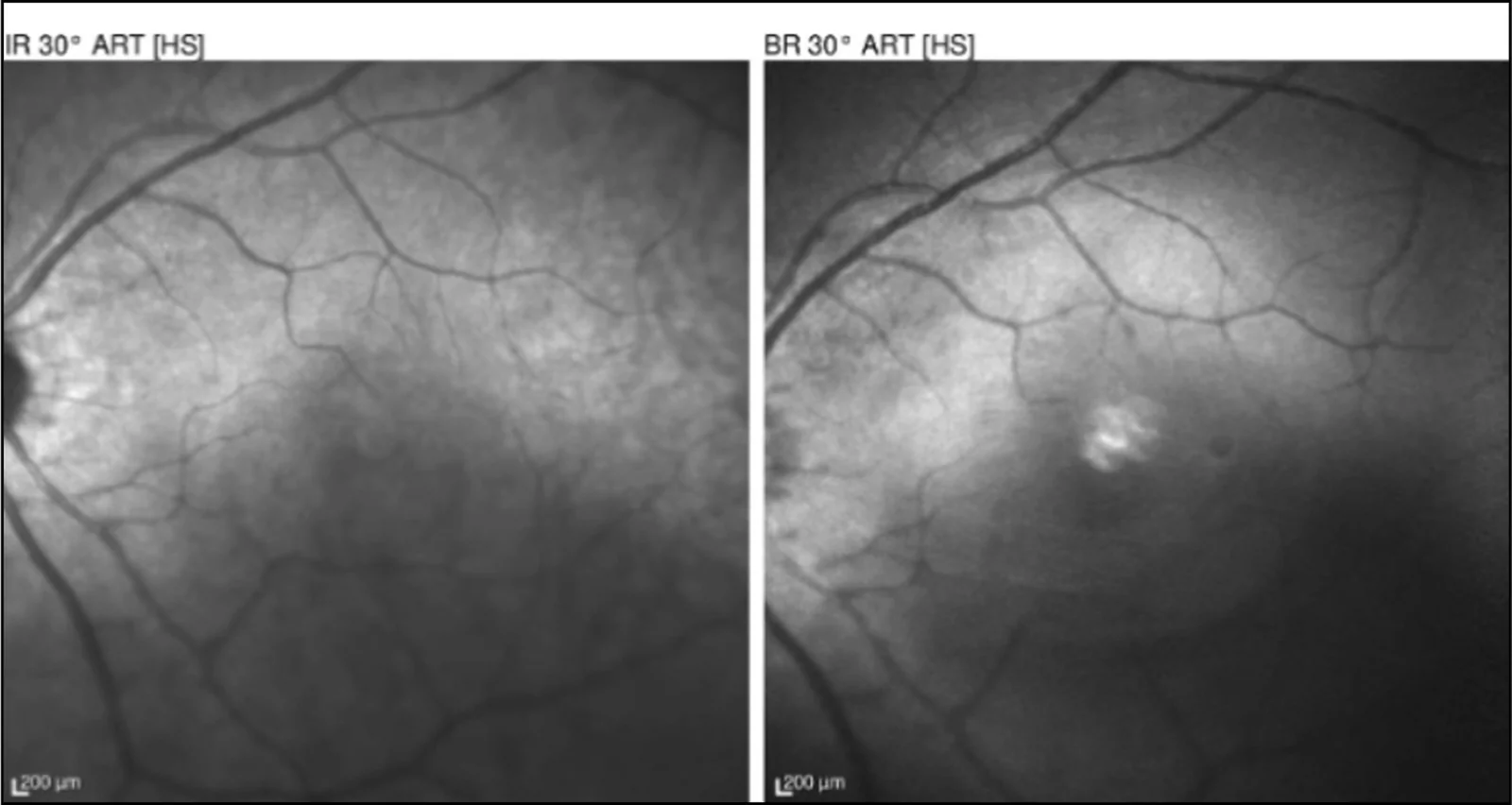
Repeat retinal imaging of affected eye at 1-week follow-up visit after initial evaluation. Compared with prior imaging, near-infrared (near-IR) and blue reflectance (BR) imaging again demonstrate a consolidated and circumscribed intravitreal opacity. The opacity demonstrates localized obscuration and blurring of the underlying macula.

## Surgical Procedure and Clinical Course

A complete pars plana vitrectomy was performed using a 25-gauge vitrectomy system (Alcon Constellation) at 20,000 cuts per minute with variable aspiration parameters. Intraoperatively, the filler appeared as a refractile, scintillating, cohesive mass freely floating within the vitreous, exerting negligible traction on the underlying retina. Vitreous was first trimmed from around the needle puncture site previously treated with laser retinopexy. Core vitrectomy was then completed, with the intravitreal filler removed alongside the native vitreous using standard cut and aspiration parameters without modification. Triamcinolone was dusted across the hyaloid face to aid visualization, after which the posterior hyaloid was removed. The anterior vitreous base was shaved with careful 360-degree scleral depression, including over areas of incidental lattice degeneration. The procedure was uncomplicated, with no intraoperative retinal breaks observed. Argon endolaser was applied at the conclusion of the case as an additional prophylactic measure against retinal tears. (See surgical highlights in online suppl. Video 1; for all online suppl. material, see https://doi.org/10.1159/cop/acaag005) The patient tolerated the procedure well and was monitored closely in the postoperative period.

On day 1 postsurgical examination, our patient’s BCVA was 20/100 and her IOP was 10 mm Hg. At subsequent postoperative follow-up, the patient’s BCVA improved to 20/30 with stable IOP and complete resolution of the previously described visual disturbances. Retinal examination and imaging demonstrated reestablishment of clarity and preservation of retinal architecture, with no evidence of retinal tears (Fig. 4). At the final postoperative visit, the patient’s BCVA was 20/20 in both eyes, and the patient reported satisfaction and relief with her outcome. The CARE Checklist has been completed by the authors for this case report, attached as online supplementary material.

**Fig. 4.**
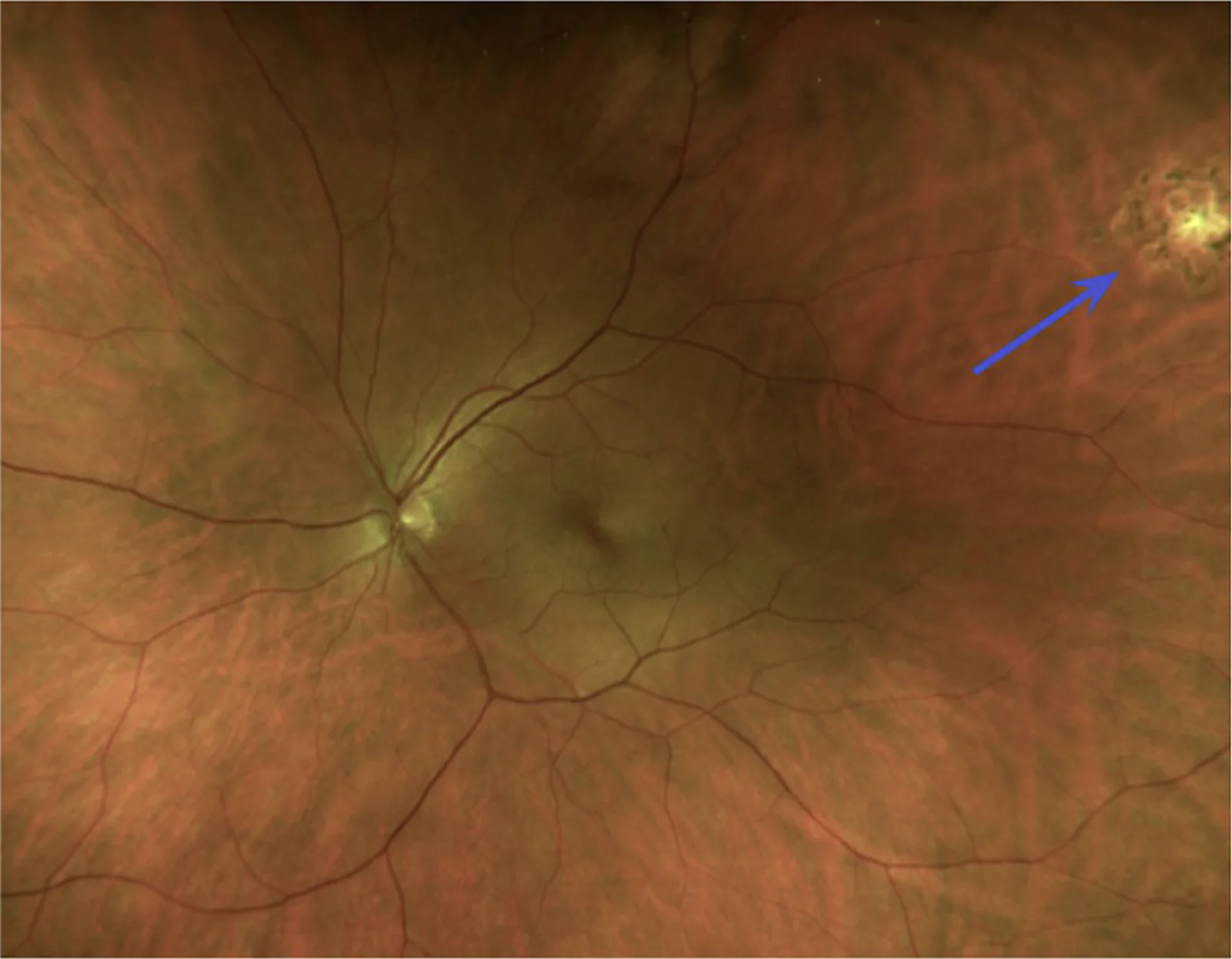
Postoperative retinal imaging. Demonstrates laser retinopexy around the puncture site (arrow) and restoration of retinal vessel clarity after removal of the previously obscuring dermal filler.

## Discussion and Future Research

When examining this case, we first sought to understand a physiologically plausible mechanism for the acute and transient vision loss the patient experienced almost immediately upon HA filler injection. We hypothesize that intravitreal filler entry induced a transient elevation in IOP, transiently reducing retinal perfusion and compressing the optic nerve. Arterial embolization was less likely the mechanism, as fluorescein angiography showed intact retinal perfusion without arterial filling defect, hemorrhage, or retinal whitening. Additionally, fundus imaging identifying a solitary retinal puncture site (Fig. 1, light blue arrow) was consistent with a single episode of mechanical globe penetration. This suggested against repeat globe entry and thus unlikely subsequent intraocular hyaluronidase injection. Partial visual recovery within 8–10 min is more consistent with IOP normalization than with hyaluronidase effect, the latter being unlikely given the filler’s persistence on imaging.

A primary management concern was how intravitreal dermal filler might interact with native ocular tissues. To contextualize this, we compared its behavior with established intraocular substances composed of HA, whose effects on the eye are well characterized as immunologically inert and of low intraocular inflammatory potential. Ophthalmic viscoelastic devices (e.g., ProVisc and Healon), composed primarily of water with approximately 1% HA, are routinely used during intraocular surgery [20]. Unlike ophthalmic viscoelastics, cosmetic HA fillers (1) are extensively cross-linked, rendering them resistant to enzymatic degradation within the vitreous, and (2) contain other inactive ingredients (e.g., phosphate buffer) whose chemical interactions with retinal tissue have not been systematically studied [21]. While overt retinal toxicity was not observed in our patient, the primary risk of retained filler likely relates to its persistence, potentially causing visual disturbance, elevated IOP, or retinal traction rather than direct chemical toxicity.

The role of hyaluronidase as a potential intravitreal treatment was briefly considered during management. Although hyaluronidase is routinely used for subcutaneous filler dissolution, we ultimately did not pursue intravitreal hyaluronidase due to limited clinical data regarding efficacy and retinal safety against highly cross-linked dermal filler within the vitreous. Though the vitreous contains endogenous hyaluronidase, Schwartz et al. [6] demonstrated that it exhibits low activity under physiologic conditions, adequate only for gradual degradation of endogenous HA. Given that Juvéderm Voluma contains 20 mg/mL of extensively cross-linked HA compared to the vitreous’s native concentration of 0.14–0.5 mg/mL, spontaneous clearance over a clinically relevant timeframe is unlikely [6, 9, 22, 23]. This conclusion is supported by our patient’s clinical course: repeat imaging at 1 week demonstrated a shifting gelatin-like mass with no reduction in filler volume or opacity, and BCVA had worsened to 20/400, indicating ongoing visual axis obstruction rather than progressive absorption. Manufacturer correspondence with Allergan Aesthetics confirmed that the cross-linking is intended to resist degradation and enhance durability and structural integrity. Though this property is desirable for dermal longevity, it may be problematic within the vitreous, where persistence risks sustained visual disturbance, elevated IOP, and retinal traction. Limited animal studies suggest intravitreal safety of hyaluronidase, but further investigation into dosing and efficacy is needed [24]. Additionally, further research is warranted to understand the long-term behavior and biocompatibility of cosmetic HA fillers within the vitreous if prompt vitrectomy is not feasible.

Prior to vitrectomy, we evaluated various rheologic properties of Juvéderm Voluma to anticipate surgical challenges. Juvéderm Voluma exhibits a high elastic modulus (G’ = 398 Pa) and comparatively low viscous component (G” = 41 Pa), indicating predominantly elastic, solid-like behavior. It has moderate cohesivity (∼40 gmf) and a maximal swelling ratio of ∼227%, maintaining structure under compression while absorbing water [25]. In contrast, native vitreous has a storage modulus of ∼1 to 10 Pa, reflecting a compliant viscoelastic material [26]. This disparity suggested Voluma would resist shear and aspiration more than vitreous, potentially necessitating technical adjustments (e.g., higher cut rates and larger lumen) for removal. Recognizing that ex vivo measurements may not reflect intravitreal behavior, we prepared a larger-gauge vitrectomy instrument and 18-gauge cannula if aspiration was difficult. Intraoperatively, however, the filler’s high cohesivity allowed removal en masse with minimal retinal interaction, consistent with its solid-like rheology.

Finally, instrument choice during periorbital filler injection warrants careful consideration, as both blunt-tipped cannulas and sharp needles carry inherent but distinct risks. Cannulas displace vessels better and require greater force to penetrate arterial walls, potentially reducing vascular complications [27]. Sharp needles allow more precise placement and aspiration but concentrate force at a single point, facilitating vascular or tissue penetration. Thus, instrument selection should be guided by injector expertise, anatomic knowledge, and procedural context [27].

## Conclusions

This case illustrates a rare but clinically significant complication of intravitreal entry of HA dermal filler. Given the prevalence of cosmetic filler use, clinicians across specialties must recognize ocular complications promptly and initiate appropriate referral and management.

Management required careful weighing of observation versus surgical intervention. Pars plana vitrectomy was selected due to the risk of progressive inflammation, persistent vitreous opacification, and tractional retinal detachment.

We hope this case serves primarily as a cautionary example emphasizing prevention through training in safe injection techniques, avoidance of high-risk injection planes, and patient education on early warning symptoms such as sudden vision loss or floaters. Our patient’s largely uncomplicated clinical and surgical course demonstrates that pars plana vitrectomy can be safely considered in carefully selected patients with intravitreal HA dermal filler and otherwise uncomplicated eyes, particularly within approximately 1 month of injection. Given the inherent limitations of a single-case report, management decisions should remain individualized.

## Statement of Ethics

This study was conducted in accordance with the principles of the Declaration of Helsinki. Ethical approval was not required for this case report in accordance with institutional policy and local and national guidelines. Written informed consent was obtained from the patient for publication of this case report and accompanying images and video.

## Conflict of Interest Statement

The authors declare no competing interests.

## Funding Sources

This research received no external financial or nonfinancial support. This research did not receive any specific grant from funding agencies in the public, commercial, or not-for-profit sectors.

## Author Contributions

Chloe J. Cohan: conceptualization, investigation, writing – original draft, review, and editing, and visualization. Jordan M. Graff: conceptualization, investigation, resources, writing – review, and editing, visualization, and supervision.

## Data Availability

The data that support the findings of this study are not publicly available due to their containing information that could compromise the privacy of the patient but are available from the corresponding author (C.J.C.) upon reasonable request.
